# A critical role for hemolysin in *Vibrio fluvialis-*induced IL-1β secretion mediated by the NLRP3 inflammasome in macrophages

**DOI:** 10.3389/fmicb.2015.00510

**Published:** 2015-05-22

**Authors:** Liqiong Song, Yuanming Huang, Meng Zhao, Zhihao Wang, Shujing Wang, Hui Sun, Biao Kan, Guangxun Meng, Weili Liang, Zhihong Ren

**Affiliations:** ^1^State Key Laboratory for Infectious Disease Prevention and Control, Collaborative Innovation Center for Diagnosis and Treatment of Infectious Diseases, National Institute for Communicable Disease Control and Prevention – Chinese Center for Disease Control and PreventionBeijing, China; ^2^Unit of Innate Immunity, Key Laboratory of Molecular Virology and Immunology, Institute Pasteur of Shanghai – Chinese Academy of SciencesShanghai, China

**Keywords:** *Vibrio fluvialis*, hemolysin, IL-1β, NLRP3 inflammasomes, macrophages

## Abstract

*Vibrio fluvialis* causes human diarrhea, but the pathogenesis is not well-studied. We hypothesized that *V. fluvialis*-secreted hemolysin (VFH) may induce IL-1β secretion through the activation of the NLRP3 inflammasome and contribute to the pathogenicity of *V. fluvialis.* To examine this possibility, we constructed VFH mutant and complement strains and demonstrated that *V. fluvialis*-induced IL-1β production and cytotoxicity in human monocytic THP-1 cells and mouse macrophages is attributed to VFH. To evaluate the role of VFH *in vivo*, we infected adult C57BL/6 mice intraperitoneally and suckling C57/B6 mice orally with various strains. The mice treated with 10^8^ CFU wild-type *V. fluvialis* or cell-free supernatant containing VFH induced significantly higher IL-1β production in peritoneal lavage fluid or in colon compared with those infected with the mutant strain, while no effect on TNF and IL-6 production was observed at day 5 or 24 h post-infection. VFH contributed to pathological changes and IL-1β release independent of colonization of *V. fluvialis* in the colon. VFH has no effect on the synthesis of pro-IL-1β, but rather it triggers the processing of pro-IL-1β into IL-1β. Furthermore, using deficient mouse strains, we verified that *V. fluvialis*-induced IL-1β is mediated through activation of Caspase-1 and the NLRP3 inflammasome *ex vivo.* Confocal microscopy suggests that VFH contributes to cathepsin B release. Furthermore, *V. fluvialis*-induced IL-1β secretion requires potassium (K^+^) eﬄux and reactive oxygen species production. Our results provide new evidence for the role of VFH in the activation of the NLRP3 inflammasome and pathogenesis in response to *V. fluvialis* infection.

**Summary Sentence:** *Vibrio fluvialis*-secreted hemolysin induces IL-1β secretion through the activation of the NLRP3 inflammasome and contributes to the pathogenicity of *V. fluvialis*.

## Introduction

*Vibrio fluvialis* causes mild to moderate dehydration, vomiting, fever, abdominal pain, diarrhea and it can be isolated from human diarrheal feces and aquatic environments (Igbinosa and Okoh, 2010; Ramamurthy et al., 2014). *V. fluvialis* infection has become an increasing public health hazard worldwide and frequently occurs in countries where the raw seafood is largely consumed (Ramamurthy et al., 2014). *V. fluvialis* accounted for 10% of *Vibrio*-caused clinical cases along the Gulf Coast in U.S. (Levine and Griffin, 1993). In China, information about the etiological characteristics of *V. fluvialis* and its epidemiology of infection are limited due to complexity in the identification and less attention in the pathogen surveillance (Liang et al., 2013).

It has been reported that *V. fluvialis* elicits intestinal fluid when fed to suckling mice and produces an array of virulence factors and toxins (Lockwood et al., 1982; Kothary et al., 2003). Cell-free culture filtrates of *V. fluvialis* strains are able to evoke distinct cytotoxic and vacuolization effects on HeLa cells (Chakraborty et al., 2005). Quorum sensing in *V. fluvialis* positively regulates production of an extracellular protease and hemolysin and affects cytotoxic activity against epithelial tissue cultures (Wang et al., 2013). Of note, the *V. fluvialis-*caused diarrhea with presence of blood is different from cholera. Thus far, the underlying mechanisms of inflammatory bloody diarrhea caused by *V. fluvialis* have not been fully defined.

The innate immune system recognizes microbial infections through a vast array of pathogen-associated molecular patterns (Takeuchi and Akira, 2010). After recognition of bacterial components, different TLRs activate signaling via the adapter MyD88, leading to the activation of the NF-κB signaling pathway. IL-1β, together with TNF-α and IL-6, are thought to be important proinflammatory mediators in initiating and maintaining the inflammatory response to pathogen and disease development during infection. IL-1β is expressed as a pro-form, which requires proteolytic cleavage for maturation. The cysteine protease Caspase-1 mediates the proteolytic processing and secretion of mature IL-1β (Fink and Cookson, 2005).

Caspase-1 is activated within inflammasomes, multiprotein complexes that also contain NLRs and the ASC. NLRP3 is essential for Caspase-1 activation in response to a variety of microbial molecules, necrotic cells and endogenous danger-associated molecules (Gurcel et al., 2006; Mariathasan et al., 2006; Kanneganti et al., 2007). Numerous pathogens activate the NLRP3 inflammasome through bacterial toxins, such as VFH (Toma et al., 2010), hemolysin of *Staphylococcus aureus* (Munoz-Planillo et al., 2009), pneumolysin (McNeela et al., 2010; Witzenrath et al., 2011), cytotoxins from *Aeromonas hydrophila* (McCoy et al., 2010). However, little is known about the host immune response to *V. fluvialis* infection. We hypothesized that upon *V. fluvialis* infection, the pore-forming VFH may induce the activation of NLRP3 inflammasome, leading to inflammatory response.

Here, we demonstrate that VFH induces cytotoxicity and the secretion of IL-1β in response to *V. fluvialis* infection in macrophages. VFH mediates the activation of the NLRP3 inflammasome and contributes to inflammatory pathology in the colon of suckling mouse orally infected with *V. fluvialis.* We further show that VFH-induced NLRP3 activation requires ROS production, cathepsin B release, and K^+^ eﬄux.

## Materials and Methods

### Bacterial Strains

Wild-type *V. fluvialis* strain 85003 used in this study was isolated from patient in China with diarrhea. Its genome sequence is available in the Sequence Read Archive under accession no. SRX397301 (Lu et al., 2014). The Δ*vfh* was constructed as previously described using allele replacement strategies (Wang et al., 2013). To construct the pUC-*vfh*, a chromosomal DNA fragment comprising the *vfh* open reading frame and its promoter sequence was amplified with the primer pair 5′-CGG AAT TCT AAG ATC ATG TCT GAA TGT-3′/5′-CGG GAT CCC GAC TGA GTT CAG CTC TCA C-3′. The amplicon was cloned as an *EcoR*I-*BamH*I fragment in pUC18 and further confirmed by DNA sequencing. The resultant plasmid, pUC-*vfh*, was introduced into *V. fluvialis* Δ*vfh* by electroporation (Marcus et al., 1990). Hemolytic phenotypes were examined using Columbia blood agar and 2% sheep erythrocytes. Unless noted otherwise, all strains were grown with aeration in brain heart infusion broth at 37°C.

### Mice and Cell Culture

C57BL/6 WT mice were obtained from Beijing Vital River Laboratory Animal Technology Co. Ltd. *Asc^-/-^* and *Nlrp3^-/-^*) mice have been described previously (Mariathasan et al., 2004; Mao et al., 2014). *Caspase-1^-^*^/^*^-^* mice were obtained from the Jackson Laboratory and crossed onto the C57BL/6 genetic background for 10 generations. These mice are also deficient for functional Caspase-11 (Kayagaki et al., 2011). All animal studies were performed in accordance with protocols approved by the Welfare & Ethical Inspection in Animal Experimentation Committee at the Chinese CDC. BMMs were prepared from the femurs and tibias of the above mice and cultured for 6 days in RPMI 1640 supplemented with 10% heat-inactivated fetal bovine serum (Life Technologies, Grand Island, NY, USA), recombinant macrophage colony-stimulating factor (20 ng/ml; R&D), 25 mmol/L HEPES, 2 mmol/L glutamine, 100 KU/L penicillin, and 100 mg/L streptomycin (all from Gibco Invitrogen, Grand Island, NY, USA). Peritoneal exudate cells were obtained by peritoneal lavage and enriched for macrophages using the method of Kumagai et al. (1979). Macrophages prepared in these methods were at least 90% pure, as assessed by expression of F4/80 using flow cytometry. The human monocytic cell line THP-1 and mouse macrophage cell line IC-21 were purchased from ATCC (TIB-202 and TIB-186). THP-1 cells were allowed to differentiate to macrophages by incubating for 48 h in the presence of 1 nM phorbol myristate acetate. All cells in this study were cultured in RPMI 1640 at a maximum concentration of 1 × 10^6^ cells/ml.

### *In Vivo* Infection

Five-days-old C57BL/6 suckling mice were infected orally with WT *V. fluvialis*, Δ*vfh V. fluvialis,* or pUC-*vfh* at 10^8^ CFU in 100 μl sterile PBS. Control mice were given PBS by gavage. Mice were sacrificed 24 h post-infection (p.i.), and the ileum and colon were homogenized or collected for staining. Diluted homogenates were plated on LB plates containing 50 μg/ml streptomycin to quantify the CFU of *V. fluvialis* colonization. A portion of the colon was fixed in 10% formaldehyde, paraffin embedded, cut into 6-mm sections, stained with hematoxylin and eosin and observed under optical microscopy. A blinded grading was performed semi-quantitatively by an outside pathologist to assess the relative degree of pathology changes. The scoring of pathology slides was assessed as follows: 0, no inflammation; 1^+^, mild inflammatory cell infiltrate with cuffing around vessels; 2^+^, increased inflammation with hyperemia; 3^+^, severe inflammation involving vascular dilation; and 4^+^, submucosal edema.

For cytokine analysis, Four-weeks-old WT C57BL/6 mice were inoculated intraperitoneally (i.p.) with 10^7^, 10^8^, or 10^9^ CFU of different bacteria in 200 μl PBS or PBS alone for control mice. Mice were sacrificed at day 5 p.i. Cytokine production in PLF was measured by ELISA using commercial kits (BD Biosciences, San Jose, CA, USA).

### *Ex Vivo* Bacterial Infection, Lactate Dehydrogenase Assay, ELISA, and ATP Assessment

Various cells were incubated with WT, Δ*vfh*, or Δ*vfh* containing pUC-*vfh* or control vector pUC18 strains of *V. fluvialis* at a MOI of 50 per cell for 3 h without LPS priming. For some experiments, the BMMs were pre-incubated for 2 h with CA-074 Me (C5857, Calbiochem, San Diego, CA, USA), Z-YVAD-FMK (Alexis Biochemicals, Lörrach, Germany), oATP (Merck, Darmstadt, Germany), *N*-acetyl-L-cysteine (NAC; A7250 Sigma-Aldrich), or KCl at the indicated concentrations. At the indicated time points, LDH activity in the culture supernatants was measured using a Cytotox96 Kit (Promega, Madison, WI, USA). The concentrations of cytokines in cell-free supernatants were quantified using commercial ELISA kits (BD Biosciences, San Jose, CA, USA). The release of ATP from the BMMs was monitored using a bioluminescence assay kit (Molecular Probes).

### Immunoblotting

Cell-free supernatants were concentrated using Amicon Ultra-4 10K Centrifugal Filter Devices (Millipore, Bedford, MA, USA). Cell extracts and concentrated supernatants were analyzed by immunoblotting. Antibodies specific for IL-1β (no. sc-52012; Santa Cruz, CA, USA), Caspase-1 (no. sc-56036), or GAPDH (no. sc-137179; Santa Cruz, CA, USA) and fluorescence-labeled secondary antibody (IRDye 800-labeled anti-rabbit IgG; 611-132-002; Rockland, Gilbertsville, PA, USA) were used, and the proteins were detected using an Odyssey Infrared Imaging System (LI-COR, Lincoln, NE, USA).

### Confocal Laser Scanning Microscopy

Mouse BMMs were left untreated or treated with bacteria. After 3 h, the extracellular bacteria were removed and the cells were fixed with 3% paraformaldehyde. The cells were then permeabilized and stained as described (Zhang et al., 2012).

### Statistical Analysis

Statistical analysis was performed using one-way ANOVA with Newman–Keuls post-testing. The correlation between ATP and IL-1β concentrations in the supernatants of BMMs co-cultured with *V. fluvialis* was assessed using the Pearson’s test and linear regression. Values of *P* < 0.05 were considered significant.

## Results

### *V. fluvialis* Colonizes Mouse Ileum and Colon and Induces IL-1β Release

**FIGURE 1:**
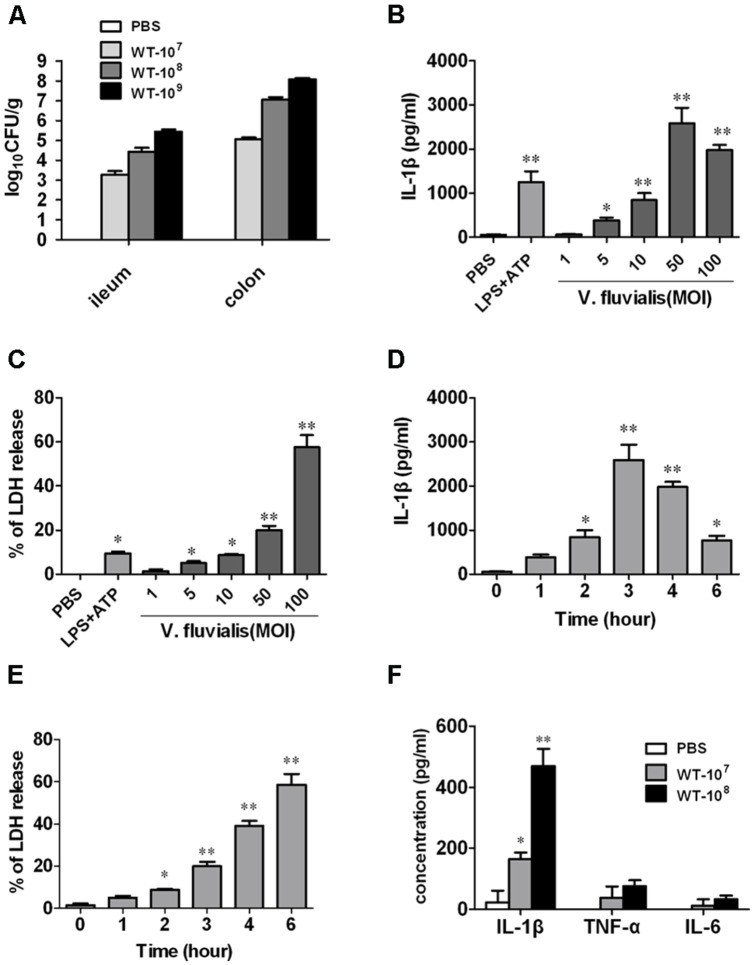
***Vibrio fluvialis* colonizes the mouse colon and induces IL-1β production and cytotoxicity in BMMs. (A)** Five-days-old C57BL/6 sucking mice were inoculated orally with 10^7^, 10^8^, or 10^9^ CFU of *V. fluvialis,* and control mice were given PBS by gavage. The ileum and colon were collected and homogenized after 24 h, and bacterial colonization was determined by plating diluted homogenates on LB plates containing 50 μg/ml streptomycin (*n* = 6 mice per group). **(B)** BMMs (1 × 10^6^) were infected with *V. fluvialis* at the indicated MOI. PBS was added as a negative control and LPS (100 ng/ml) + ATP (5 mM) was added as a positive control. After 3 h treatment, IL-1β production in culture supernatants was analyzed by ELISA. **(C)** BMMs (1 × 10^6^) were infected with *V. fluvialis* at different doses. The release of LDH was measured as an indicator of cytotoxicity. **(D)** BMMs (1 × 10^6^) were treated with *V. fluvialis* (MOI, 50) and supernatants were collected at different time points to detect IL-1β by ELISA. **(E)** BMMs (1 × 10^6^) were treated with *V. fluvialis* (MOI, 50) and supernatants were collected at different time points to assess LDH for cytotoxicity. **(F)** Four-weeks-old C57BL/6 mice were injected i.p. with 10^7^ or 10^8^ CFU of *V. fluvialis,* and at day 5 p.i. PLF was harvested for IL-1β, TNF-α and IL-6 measurement by ELISA (*n* = 6 mice per group). ELISA and cytotoxicity data represent mean ± SD of six independent mice. ^∗^*P* < 0.05; ^∗∗^*P* < 0.01.

To determine whether *V. fluvialis* can infect and productively colonize mice, we challenged suckling mice orally with different doses of *V. fluvialis* for 24 h. Our results show that *V. fluvialis* colonized in both ileum and colon in a dose-dependent manner (**Figure 1A**).

To assess the macrophage response to *V. fluvialis*, we treated mouse BMMs with *V. fluvialis* at different MOI. *V. fluvialis* induced IL-1β secretion and cytotoxicity in a dose-dependent manner, with peak levels of IL-1β production at 50 MOI, which caused 20% cytotoxicity (**Figures 1B,C**). At this MOI, *V. fluvialis* also promoted a time-dependent increase in IL-1β production, which peaked at 3 h p.i., as well as an increase in cytotoxicity (**Figures 1D,E**).

To assess the proinflammatory response induced by *V. fluvialis in vivo,* 4-weeks-old C57BL/6 mice were injected i.p. with 10^7^ or 10^8^ CFU of *V. fluvialis* and the cytokines levels in PLF were measured by ELISA at day 5 p.i. The results showed that the mice injected with *V. fluvialis* induced higher levels of IL-1β secretion, though the levels of TNF-α and IL-6 did not change significantly (**Figure 1F**). These results verify that *V. fluvialis* can colonize the mouse colon and specifically activate IL-1β.

### VFH Plays a Critical Role in *V. fluvialis*-Induced IL-1β Release and Cytotoxicity *Ex Vivo*

**FIGURE 2:**
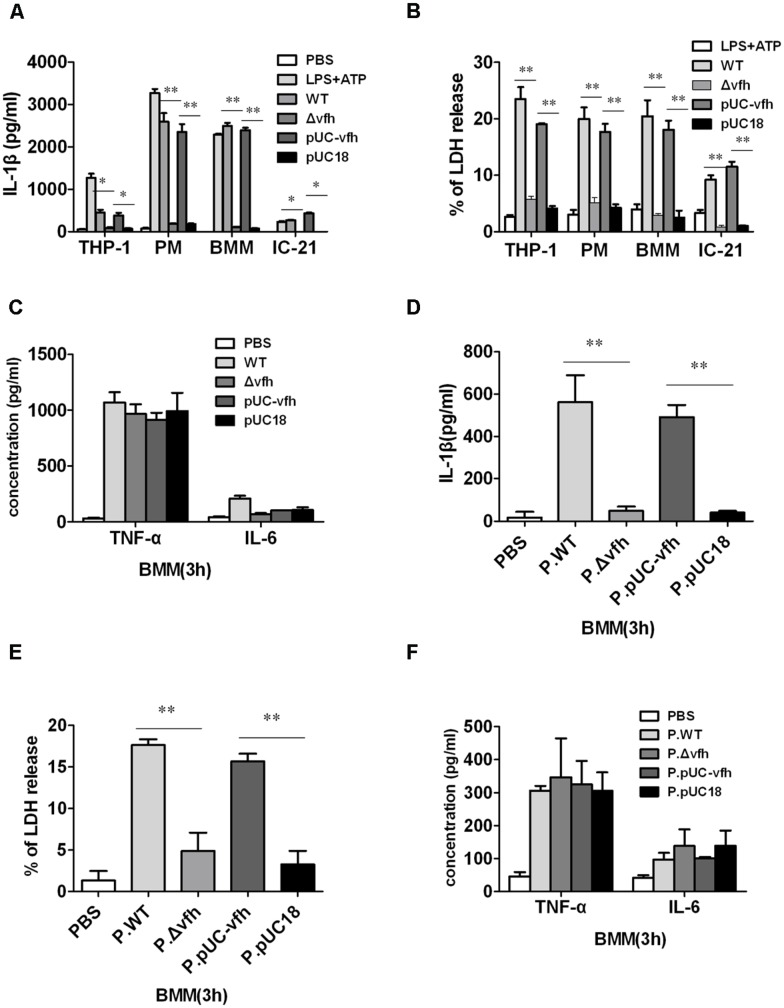
***Vibrio fluvialis*-secreted hemolysin triggers IL-1β production and cytotoxicity *ex vivo.* (A–C)** 1 × 10^6^ human monocytic THP-1 cells, mouse peritoneal macrophages (PMs), mouse BMM, or mouse macrophage IC-21 cells were infected at MOI 50 for 3 h with WT *V. fluvialis* (WT), *V.* fluvialis with a mutated VFH gene (Δ*vfh)*, Δ*vfh* complemented with a VFH gene (pUC-*vfh)*, or Δ*vfh* complemented with an empty vector (pUC18). The levels of IL-1β **(A)**, IL-6 and TNF-α **(C)** in the supernatants were determined by ELISA, and the levels of LDH release **(B)** was quantified using the Cytotox96 Kit (Promega). **(D–F)** BMMs were treated for 3 h with PBS or supernatant from overnight culture of WT, Δ*vfh*, pUC-*vfh,* or pUC18 strains in PBS (designated as P.WT, P.Δ*vfh*, P.pUC-*vfh,* and P.pUC18). The levels of IL-1β **(D)**, IL-6 and TNF-α **(F)** in the supernatants were determined by ELISA, and the LDH release **(E)** was quantified using the Cytotox96 Kit. Results represent mean ± SD of at least three independent experiments. ^∗^*P* < 0.05; ^∗∗^*P* < 0.01.

To determine which component of *V. fluvialis* mediates *V. fluvialis*-induced IL-1β release and cytotoxicity in various cells, we constructed an isogenic mutant (Δ*vfh*) and complement strain of VFH (pUC-*vfh*). WT and pUC-*vfh* strains induced significantly higher levels of IL-1β and cytotoxicity compared with the Δ*vfh* strains in human THP-1 cells, mouse IC-21 cells, mouse peritoneal macrophages (PMs), and BMMs at 3 h p.i. (**Figures 2A,B**). In contrast, VFH did not contribute to the IL-6 and TNF-α production induced by *V. fluvialis* (**Figure 2C**).

To confirm that the secreted form of VFH can trigger IL-1β release and cytotoxicity, we treated BMMs with different supernatants of overnight cultures from WT, Δ*vfh,* and pUC-*vfh* strains for 3 h. The supernatant of WT and pUC-*vfh* induced significantly higher levels of IL-1β production and cytotoxicity in BMMs than the supernatant of Δ*vfh* (**Figures 2D,E**), though the production of TNF-α and IL-6 was comparable for all supernatants (**Figure 2F**). These results suggest that VFH has an essential and specific role in the release of IL-1β and cytotoxicity in human and mouse monocyte/macrophage cell lines and primary mouse macrophages.

### VFH Plays a Critical Role in *V. fluvialis*-Induced IL-1β Release and Cytotoxicity *In Vivo*

**FIGURE 3:**
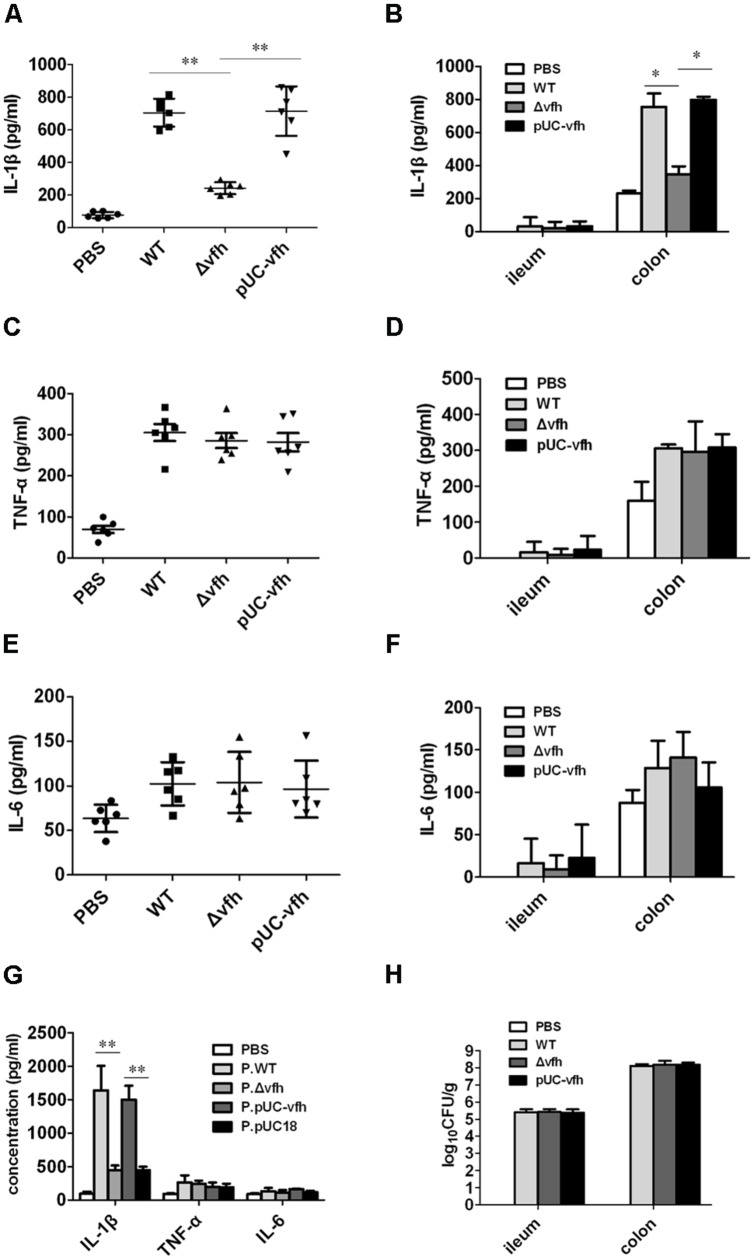
***Vibrio fluvialis*-secreted hemolysin triggers IL-1β production and cytotoxicity *in vivo*. (A,C,E)** C57BL/6 mice were injected i.p. with PBS as control or 10^8^ CFU WT, Δ*vfh*, or pUC-*vfh*. Mice (*n* = 6) were sacrificed 5 days after injection, and the IL-1β **(A)**, TNF **(C)** and IL-6 **(E)** in PLF was measured by ELISA. **(B,D,F)** Five-days-old sucking mice were infected orally with PBS as a control or 10^8^ CFU WT, Δ*vfh*, or pUC-*vfh*. Mice (*n* = 6) were sacrificed 24 h p.i. and the ileum and colon were homogenized. The production of IL-1β **(B)**, TNF **(D)** and IL-6 **(F)** in homogenate was assayed by ELISA. **(G)** Five-days-old sucking mice (*n* = 6) were treated by gavage with cell-free supernatant from overnight culture of various strains. The level of IL-1β, TNF, and IL-6 in homogenates of colon at 24 h p.i. were measured by ELISA. **(H)** The *V. fluvialis* colonization in ileum and colon was quantified by plating the diluted homogenates on LB plates containing 50 μg/ml streptomycin. Results represent the mean + SD of triplicates. ^∗^*P* < 0.05; ^∗∗^*P* < 0.01.

To evaluate the role of VFH in inflammatory response, bacterial colonization, and pathology change induced by *V. fluvialis in vivo*, we infected adult mice i.p. with 10^8^ CFU WT, Δ*vfh,* and pUC-*vfh* strains and measured the cytokines in PLF at day 5 p.i. The mice injected with WT and pUC-*vfh* strains induced significantly more IL-1β than those injected with Δ*vfh* (**Figure 3A**). The VFH-dependent IL-1β induction was confirmed in colon using a suckling mouse model (**Figure 3B**). In contrast to the results for IL-1β, no difference among the strains was observed for TNF-α (**Figures 3C,D**) or IL-6 (**Figures 3E,F**).

To verify the *in vivo* function of soluble VHF, we inoculated suckling mice with supernatants from overnight cultures of various strains. The supernatant from WT and pUC-*vfh* compared with Δ*vfh* induced significantly higher levels of IL-1β release in colon without affecting TNF and IL-6 production (**Figure 3G**). Furthermore, we did not observe a difference in bacterial colonization in the colon after mice were challenged with three strains (**Figure 3H**), suggesting that VFH contributes to IL-1β release independent of colonization of *V. fluvialis*. Taken together, these findings confirm that VFH secreted by *V. fluvialis* specifically mediates IL-1β release upon *V. fluvialis* challenge.

### VFH of *V. fluvialis* Induces Histopathological Lesions in the Colon

**FIGURE 4:**
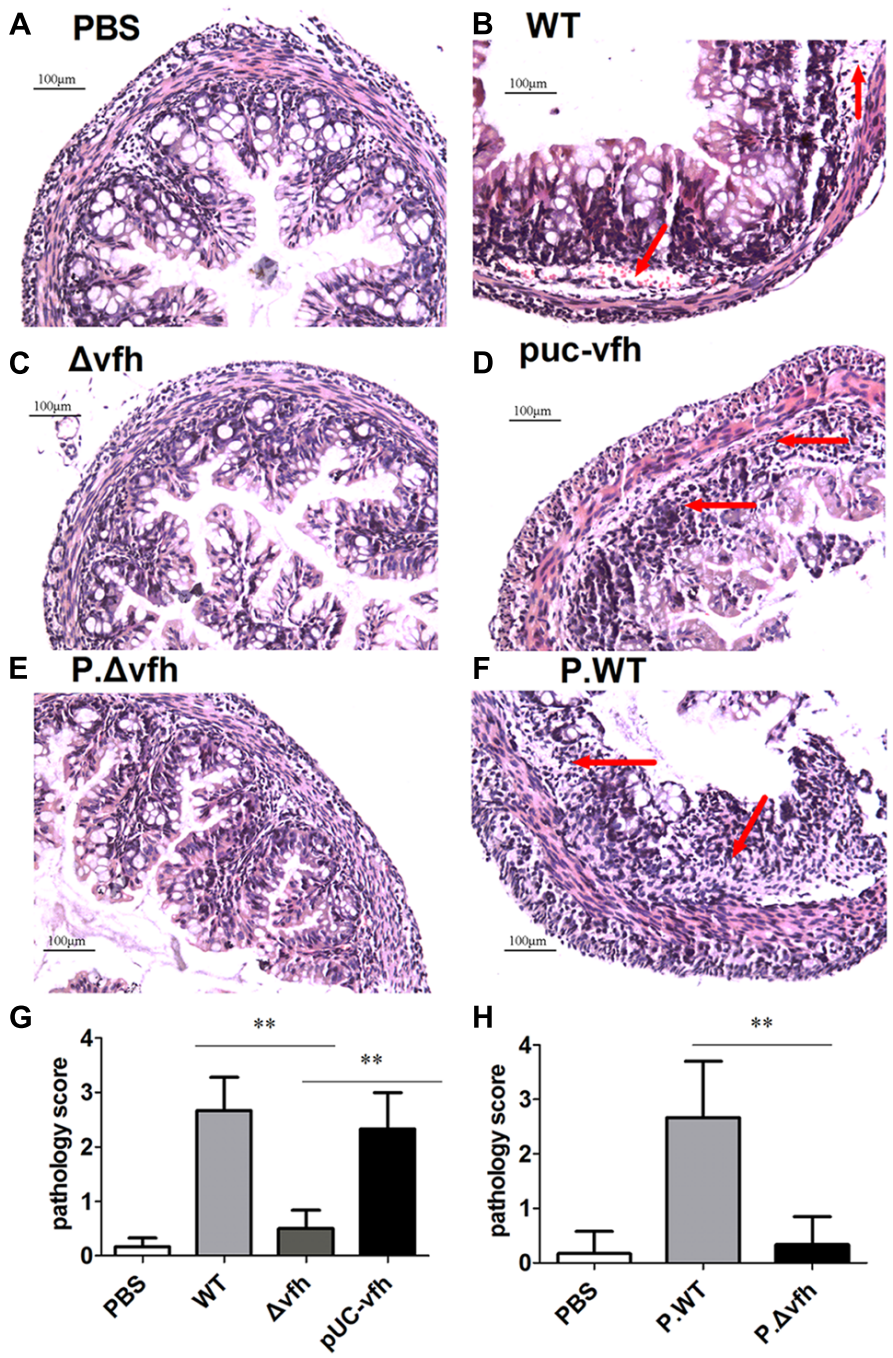
**Histopathological analysis of colon lesions.** Five days C57 BL/6 sucking mice were inoculated orally with various strains or supernatant from cell-free overnight culture of various strain of *V. fluvialis*. Colons were collected 24 h p.i., and the specimens were fixed, embedded, cut, and stained with H.E. **(A)** Negative control mouse treated with PBS. **(B)** Infection with WT *V. fluvialis*. **(C)** Infection with the mutant strain Δ*vfh*. **(D)** Infection with complemented mutant strain pUC-*vfh*. **(E)** Treatment with supernatant from Δ*vfh*. **(F)** Treatment with supernatant from WT. **(G)** The pathology scoring in mice treated with WT or Δ*vfh* or pUC-*vfh* strain. **(H)** The pathology scoring in mice treated with supernatant from Δ*vfh* and WT. The result represent mean ± SD of six independent mice. ^∗^*P* < 0.05; ^∗∗^*P* < 0.01 and are shown as 200 magnification times, **(A–H)** 200×.

To characterize the inflammatory histopathology in colon caused by *V. fluvialis* infection, we inoculated five-days-old sucking mice with various strains or cell-free supernatant from overnight culture with various strains by gavage. At 24 h p.i., there were no lesions or obvious abnormalities in the negative control mice treated with PBS (**Figure 4A**); however, WT *V. fluvialis* caused submucosal edema, vascular dilation, hyperemia, and inflammatory cell infiltration as indicated by red arrow (**Figure 4B**). Mice infected with Δ*vfh* showed similar pathology to the control mice (**Figure 4C**), whereas the pUC-*vfh* strain induced severe lesions including obvious epithelial shedding, glandular structure damage and inflammatory cell infiltration as pointed by arrow (**Figure 4D**). The effects of the Δ*vfh* and WT supernatants were similar to the effects of the Δ*vfh* and WT strains. (**Figures 4E,F**). The summary data showed that the mice treated with WT *V. fluvialis* or supernatants of WT culture had significantly higher colon pathology scores than did those mice treated with Δ*vfh* or supernatants of Δ*vfh* (**Figures 4G,H**). All these data verify that VFH contributes to the pathogenicity of *V. fluvialis* in the colon.

### VFH Triggers IL-1β via Activation of Caspase-1 and the NLRP3 Inflammasome

**FIGURE 5:**
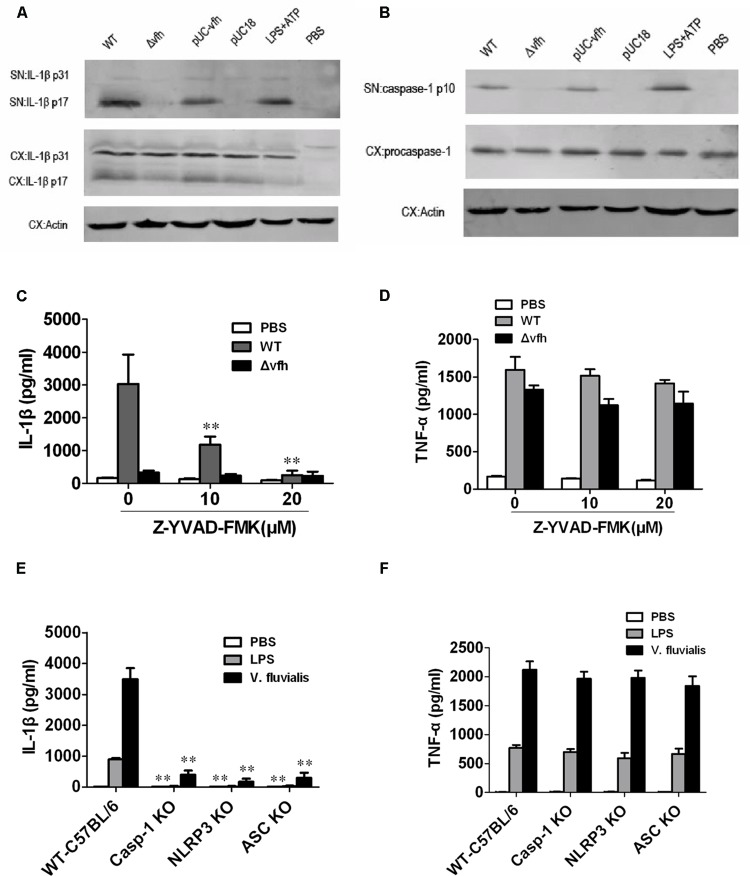
***Vibrio fluvialis*-secreted hemolysin-induced IL-1β secretion is dependent on Caspase-1 and NLRP3 inflammasome activation in mouse BMM.** BMMs (1 × 10^6^) were infected with WT, Δ*vfh*, pUC-*vfh,* or pUC18 *V. fluvialis* strains at MOI 50 for 3 h or were incubated with PBS (negative control) or LPS + ATP (positive control). **(A)** Amounts of IL-1β p17 and p31 in supernatant (SN) and cell extract (CX) were visualized by Western blotting. **(B)** Amounts of active Caspase-1 p10 and procaspase-1 and in SN and CX were visualized by Western blotting. **(C,D)** The levels of IL-1β secretion **(C)** and TNF-α secretion **(D)** after infection of BMMs in the presence of the Caspase-1 inhibitor Z-YVAD-FMK were measured by ELISA. **(E,F)** The levels of IL-1β secretion **(E)** and TNF-α secretion **(F)** were measured in BMMs isolated from WT C57 B/6 or *Caspase-1^-^*^/^*^-^*, *Nlrp3^-^*^/^*^-^*, or *Asc^-^*^/^*^-^* mouse after infection with *V. fluvialis*. Values represent the mean + SD of triplicates and are representative of three independent experiments. ^∗^*P* < 0.05; ^∗∗^*P* < 0.01.

To study the mechanism of VFH-triggered IL-1β, we measured mRNA expression level of IL-1β induced by different strains and found that VFH had no effect on the mRNA expression of IL-1β (Supplementary Figure S1). We further examined the production of inactive pro-IL-1β (p31) and mature active IL-1β (p17) in supernatants and cell lysates using immunoblotting after infection. WT and pUC-*vfh* induced significantly higher levels of active mature IL-1β in the supernatants than Δ*vfh* did, which is consistent with the ELISA results; however, all strains induced similar levels of biologically inactive pro-IL-1β in cell lysates (**Figure 5A**). Furthermore, the secretion of p10 subunit of Caspase-1 was evident in the supernatants of BMMs infected with WT and pUC-*vfh* but not in negative control cells or those treated with the Δ*vfh* strain (**Figure 5B**). These results verify that the induction of IL-1β by VFH occurs at the level of inflammasome processing. To verify the role of Caspase-1, we repeated the ELISA experiments in the presence of the Caspase-1 inhibitor Z-YVAD-FMK. Z-YVAD-FMK dramatically inhibited IL-1β secretion (**Figure 5C**), but not TNF-α secretion (**Figure 5D**). Furthermore, *V. fluvialis*-induced IL-1β was abolished for BMMs isolated from *Caspase-1^-^*^/^*^-^*, *Nlrp3^-^*^/^*^-^*, or *Asc^-^*^/^*^-^* mice, though the release of TNF-α was not affected (**Figures 5E,F**). Giving the fact that VFH did not obviously affect message level of IL-1β and it indeed induced Caspase-1 activation and caused more mature IL-1β secreted, we conclude that VFH may affect IL-1β production at the level of IL-1β processing or secreting.

### Cathepsin B Release Contributes to *V. fluvialis*-Induced-IL-1β Secretion in Mouse BMMs

**FIGURE 6:**
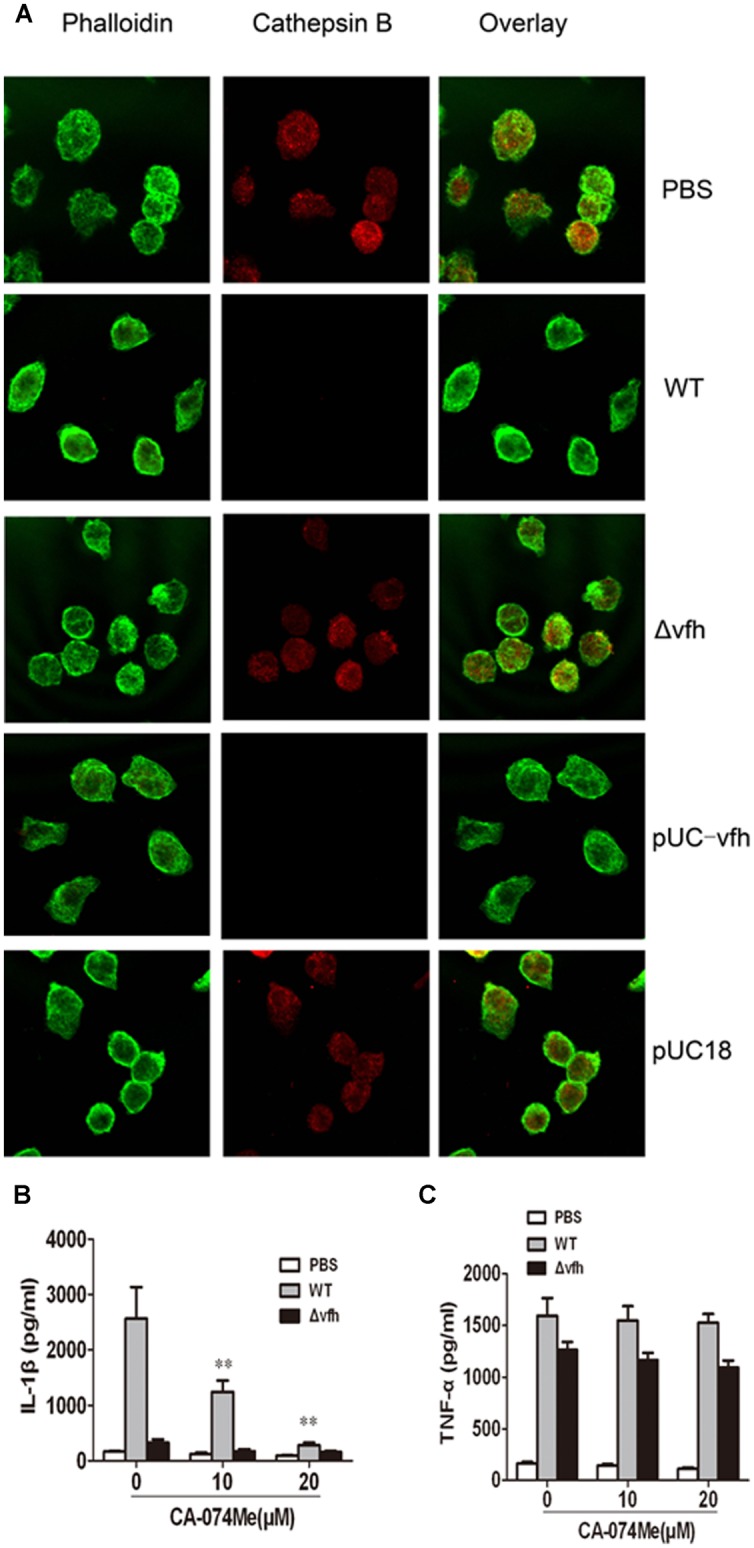
**Cathepsin B is involved in IL-1β secretion in BMMs upon *V. fluvialis* infection. (A)** BMMs were left untreated or were infected with WT, Δ*vfh*, pUC-*vfh,* or pUC18 *V. fluvialis* strains for 2 h. Cathepsin B was visualized by confocal laser scanning microscopy using a specific Ab. Actin was stained with phalloidin AF488. **(B,C)** BMMs were treated with cathepsin B inhibitor CA-074Me at the indicated concentrations for 1 h before infection with WT or Δ*vfh* for 3 h. IL-1β **(B)** and TNF-α **(C)** in the supernatants were quantified by ELISA. Results represent mean ± SD of three independent experiments. ^∗^*P* < 0.05; ^∗∗^*P* < 0.01.

To determine whether cathepsin B is activated and released from cells upon *V. fluvialis*-induced IL-1β release, we performed immunofluorescence staining and confocal microscopy of BMMs. Uninfected cells showed punctate staining, which was obviously diminished in WT-infected cells and pUC-Δ*vfh*-infected cells, but was apparent in Δ*vfh*-infected cells. These results suggest that VFH contributes to cathepsin B release from phagoendosomes to the cytosol (**Figure 6A**).

To verify these findings, we assessed the effects of the cathepsin B inhibitor CA-074Me on *V. fluvialis*-induced IL-1β secretion. CA-074Me significantly attenuated IL-1β production in BMMs infected with WT *V. fluvialis* (**Figure 6B**) but had no effect on TNF-α secretion (**Figure 6C**).

### *V. fluvialis*-Induced Activation of the NLRP3 Inflammasome in BMMs Requires Potassium (K^+^) Eﬄux, and ROS Production

**FIGURE 7:**
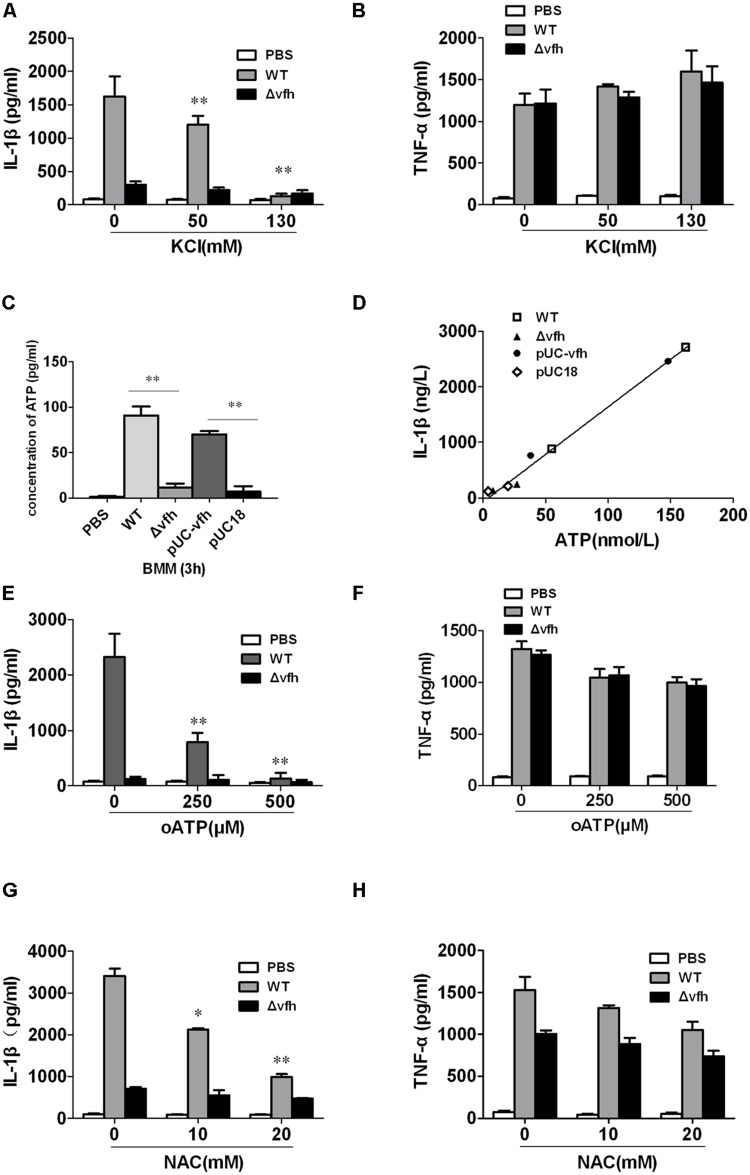
***Vibrio fluvialis* hemolysin-induced IL-1β production in BMMs requires ATP signaling, K^+^-eﬄux and ROS generation. (A,B)** BMMs were infected with *V. fluvialis* WT or Δ*vfh* in the absence or presence of KCl. IL-1β **(A)** and TNF-α **(B)** in the supernatants were determined at 3 h p.i. using ELISA. **(C)** The release of ATP from the BMMs infected with various strains was monitored using a bioluminescence assay kit. **(D)** The correlation between extracellular ATP release and IL-1β secretion is plotted for BMMs infected with different *V. fluvialis* strains at 3 h. **(E–H)** BMMs were infected with *V. fluvialis* WT or Δ*vfh* in the absence or presence of oxidized ATP (oATP; **E,F**) or *N*-acetyl-L-cysteine (NAC; **G,H**). IL-1β and TNF-α in the supernatants were determined at 3 h p.i. using ELISA. Results represent mean ± SD of three independent experiments. ^∗^*P* < 0.05; ^∗∗^*P* < 0.01.

To investigate the role of K^+^ eﬄux in *V. fluvialis*-induced IL-1β production in BMMs, we added KCl to the cell culture medium to block K^+^ eﬄux prior to infection with *V. fluvialis.* KCl almost completely inhibited IL-1β production (**Figure 7A**), though no effects on TNF levels were observed (**Figure 7B**). Because extracellular ATP has been shown to trigger K^+^ eﬄux and NLRP3 activation via the ATP receptor P2X7 (Franchi et al., 2007), we sought to determine whether VFH affects extracellular ATP release. The results show that the WT and pUC-*vfh* strains induced significantly higher levels of ATP release into the supernatant than the Δ*vfh* strain did (**Figure 7C**). Furthermore, there was a significantly positive correlation between ATP release and IL-1β secretion in BMMs (**Figure 7D**). To further study whether *V. fluvialis* exerts its effect through the ATP receptor P2X7, we tested the effects of the P2X7R inhibitor, oATP, on IL-1β release. oATP significantly reduced *V. fluvialis*-induced IL-1β levels (**Figure 7E**) although it had no effect on TNF-α production (**Figure 7F**). Finally, to determine the role of ROS in *V. fluvialis*-induced NLRP3 inflammasome activation, we pretreated BMMs with the ROS inhibitor NAC. NAC impaired *V. fluvialis*-induced IL-1β release in a dose-dependent manner (**Figure 7G**). NAC also reduced the production of TNF-α to a lesser extent (**Figure 7H**), which is different from the effects of KCl and oATP. Collectively, these findings suggest that *V. fluvialis* induction of IL-1β involves K^+^ eﬄux, extracellular ATP release, and the production of ROS.

## Discussion

Prior to this study, little was known regarding the mechanism of inflammatory response induced by *V. fluvialis*. Previous work identified VFH as a virulence factor of *V. fluvialis* for its role in lysing erythrocytes of various host origin and activating fluid accumulation (Kothary et al., 2003). VFH is a member of the pore-forming toxin family, which has a wide spectrum of cytocidal activity. It has been reported that bacterial hemolysins, for example, listeriolysin O of *Listeria monocytogenes* (Meixenberger et al., 2010), hemolytic cytolysin pneumolysin of *Staphylococcus pneumonia* (Witzenrath et al., 2011) and hemolysin of *S. aureus* (Kebaier et al., 2012), can induce the release of IL-1β through activation of NLRP3 inflammasome. We compared the amino sequence of these toxins and found out that the amino acid similarity between VFH and pneumolysin of *S. pneumonia* or listeriolysin O of *L. monocytogenes* or Staphylococcal α-hemolysin is 23, 27, or 24%, respectively. Of note, the amino similarity between VFH and *Vibrio cholera* hemolysin is 72%. However, it is not known whether VFH contributes to inflammation response during *V. fluvialis* infection. The presented data in this manuscript not only provides information that VFH induces IL-1β release dependent on NLRP3, but also demonstrate that VFH plays a key role in a suckling mouse model in contributing to host colon pathology and inflammatory response upon *V. fluvialis* infection, suggesting that VFH may be an important virulence factor of *V. fluvialis* causing inflammatory diarrhea in humans.

In this study, we unexpectedly found that VFH induced greater amount of IL-1β in colon but no detectable level of IL-1β in ileum (data not shown) and that IL-1β production was independent of colonization of *V. fluvialis* using the suckling mouse model. One possible explanation is that there may be some difference in the number of IL-1β-producing cells (like macrophages) or in their function of producing cytokines in response to *V. fluvialis* stimulation between ileum and colon. There is plenty of microbiota colonization in colon so the inflammatory response by innate immune cells in colon maybe functionally different from the cells in ileum. The exact mechanisms underlying the different inflammatory response induced by *V. fluvialis* between ileum and colon deserve further investigation. We agree that the inflammation triggered by VFH is a “double-edged sword” for the host. On the one hand, VFH-induced IL-1β, a key cytokine in the host’s immune response, contributes to controlling of the bacteria infection. On the other hand, if too much inflammation was induced, it will cause damage to the host cells and tissues and to evade certain defense mechanisms resulting in the severe inflammatory pathology changes.

We demonstrated that VFH can specifically trigger IL-1β but not TNF-α and IL-6 secretion. IL-1β is produced in a two-step process with the first step involving the generation of the biologically inactive precursor pro–IL-1β, typically in response to TLR activation (Bauernfeind et al., 2009; Franchi et al., 2009), and the second step involving pro–IL-1β cleavage by Caspase-1 into an active cytokine. The activation of Caspase-1 is controlled by inflammasomes. Recent studies have shown that many bacterial toxins, including the hemolysins of various other bacterial species trigger NLRP3 inflammasome activation (Harder et al., 2009; Munoz-Planillo et al., 2009; McNeela et al., 2010; Meixenberger et al., 2010; Toma et al., 2010; Holzinger et al., 2012; Zhang et al., 2012). Our data show that VFH did not affect the expression of IL-1β mRNA and pro-IL-1β synthesis but had an effect on activating the NLRP3 inflammasome and inducing the mature active IL-1β, therefore we propose that VFH affects IL-1β production at the level of IL-1β processing or secretion. The mature biological active IL-1β plays a critical role in host immune response. We confirmed the effect of VFH on secreted mature IL-1β and therefore have added a new member to the list of bacterial pore-forming toxins triggering IL-1β via Caspase-1 and NLRP3 inflammasome activation.

Cellular stimulation triggers ATP release and subsequently activates purinergic receptors in an autocrine and/or paracrine manner (Piccini et al., 2008), although certain non-nucleotide inflammasome activators may interact directly with purinergic receptors, which does not depend on ATP release (Babelova et al., 2009). In the present study, we demonstrated that VHF contributes to the ATP release into supernatant and that P2X7R signaling is involved in the inflammasome activation induced by *V. fluvialis*. A recent study has shown that the eﬄux of K^+^ is responsible for the maturation of pro-IL-1β (Petrilli et al., 2007). K^+^ eﬄux occurs upon the engagement of extracellular ATP with the P2X7R or directly through bacterial pore-forming toxins (Munoz-Planillo et al., 2013). Some bacterial pore-forming toxins were reported to cause K^+^ eﬄux by permeabilizing the plasma membrane (Walev et al., 1993; Fiser et al., 2012), which activates Nlrp3 independently of P2X7R (Harder et al., 2009). Therefore, we propose that VFH may induce extracellular ATP release, which results in K^+^ eﬄux via P2X7R indirectly. It has yet to be determined how K^+^ eﬄux is induced by other classes of inflammasome activators. More recently, calcium has been shown to play a role in NLRP3 inflammasome activation (Lee et al., 2012; Rossol et al., 2012). Whether migration of calcium is required in *V. fluvialis*-induced activation of the NLRP3 inflammasome warrants further investigation.

NLR family pyrin domain containing 3 activators induce lysosomal damage, which leads to the release of cathepsin B into the cytosol. The lysosomal proteases, in turn, could either degrade a putative NLRP3 inhibitor or cleave a substrate in the cytosol that would generate a NLRP3 ligand (Halle et al., 2008). Our results demonstrate that VFH significantly contributes to cathepsin B release and that an inhibitor of cathepsin B causes a modest decrease in IL-1β production in BMMs. It remains to be determined how cathepsin B release plays its role in VFH-induced NLRP3 activation in BMMs. Furthermore, our data show that ROS production is required for *V. fluvialis*-triggered NLRP3 activation. Consistent with these results, previous studies have shown that ROS inhibitors such as DPI and NAC strikingly inhibit IL-1β and IL-8 production in mouse macrophages (Bauernfeind et al., 2011; Mao et al., 2014).

In summary, this study demonstrates that VFH plays a key role in mediating IL-1β secretion and histopathology in the colon during *V. fluvialis* infection *in vivo.* Our data also suggest that VFH-mediated activation of the NLRP3 inflammasome is critically involved in the proinflammatory response upon *V. fluvialis* infection. Several signaling pathways are involved in NLRP3 activation, though K^+^ eﬄux appears to play a more important role in *V. fluvialis*-induced IL-1β production than ATP and cathepsin B release do in this study. These findings are helpful for understanding the role of VFH in the pathogenesis of *V. fluvialis*.

## Author Contributions

LS and ZR conceived and designed the experiments. LS, YH, MZ, ZW, HS, and SW performed the experiments. LS and ZW analyzed the data. WL, BK, and GM discussed the results. LS and ZR wrote the paper.

## Conflict of Interest Statement

The authors declare that the research was conducted in the absence of any commercial or financial relationships that could be construed as a potential conflict of interest.

## Supplementary Material

Figure S1 **mRNA expression of IL-1β in BMMs induced by *Vibrio fluvialis*.** BMMs were treated with WT, Δ*vfh*, pUC-*vfh,* or pUC18 *V. fluvialis* strains at MOI 50 for 3 h or were incubated with PBS. Cells were lysed over 3 h post-infection and mRNA expression of IL-1β was analyzed using RT-PCR. Results represent mean ± SD of three independent experiments.

## Abbreviations

ASC: adapter molecule
*Asc^-^*^/^*^-^*: Asc-deficient
BMMs: mouse bone marrow derived macrophages
*Caspase-1^-^*^/^*^-^*: Caspase-1-deficient
i.p.: intraperitoneally
LDH: lactate dehydrogenase
MOI: multiplicity of infection
NLRs: nod-like receptors
*Nlrp3^-^*^/^*^-^*: Nlrp3-deficient
NLRP3: NLR family pyrin domain containing 3
PLF: peritoneal lavage fluid
pUC-*vfh*: Δ*vfh V. fluvialis* with complement plasmid
ROS: reactive oxygen species
VFH: *V. fluvialis*-secreted hemolysin
Δ*vfh*: isogenic *vfh* mutant
WT: wild-type.
