# Supplementary figures and images for: A critical role for hemolysin in Vibrio fluvialis-induced IL-1β secretion mediated by the NLRP3 inflammasome in macrophages
Source: Front Microbiol. 2015 May 22;6:510. doi: 10.3389/fmicb.2015.00510 (PMC4440915; doi:10.3389/fmicb.2015.00510)

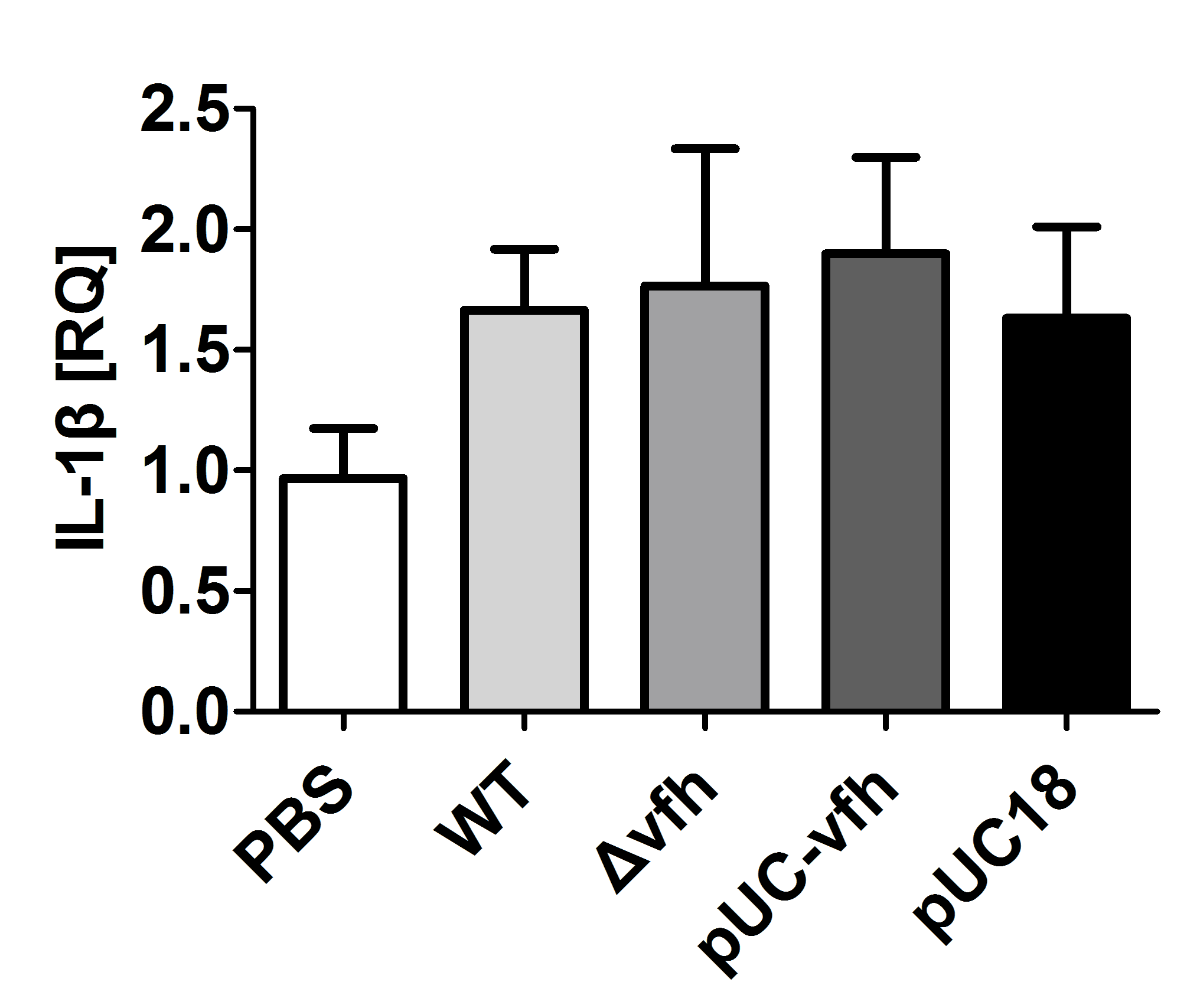

Supplement: Figure S1 — mRNA expression of IL-1β in BMMs induced by Vibrio fluvialis. BMMs were treated with WT, Δvfh, pUC-vfh, or pUC18 V. fluvialis strains at MOI 50 for 3 h or were incubated with PBS. Cells were lysed over 3 h post-infection and mRNA expression of IL-1β was analyzed using RT-PCR. Results represent mean ± SD of three independent experiments. [file Image_1.TIF]
